# Diel Variability in Seawater pH Relates to Calcification and Benthic Community Structure on Coral Reefs

**DOI:** 10.1371/journal.pone.0043843

**Published:** 2012-08-28

**Authors:** Nichole N. Price, Todd R. Martz, Russell E. Brainard, Jennifer E. Smith

**Affiliations:** 1 Scripps Institution of Oceanography, University of California San Diego, La Jolla, California, United States of America; 2 Coral Reef Ecosystem Division, Pacific Islands Fisheries Science Center, National Oceanic and Atmospheric Administration, Honolulu, Hawaii, United States of America; National Institute of Water & Atmospheric Research, New Zealand

## Abstract

Community structure and assembly are determined in part by environmental heterogeneity. While reef-building corals respond negatively to warming (i.e. bleaching events) and ocean acidification (OA), the extent of present-day natural variability in pH on shallow reefs and ecological consequences for benthic assemblages is unknown. We documented high resolution temporal patterns in temperature and pH from three reefs in the central Pacific and examined how these data relate to community development and net accretion rates of early successional benthic organisms. These reefs experienced substantial diel fluctuations in temperature (0.78°C) and pH (>0.2) similar to the magnitude of ‘warming’ and ‘acidification’ expected over the next century. Where daily pH within the benthic boundary layer failed to exceed pelagic climatological seasonal lows, net accretion was slower and fleshy, non-calcifying benthic organisms dominated space. Thus, key aspects of coral reef ecosystem structure and function are presently related to natural diurnal variability in pH.

## Introduction

Awareness of the potential threat of ocean acidification (OA) to marine organisms has risen sharply over the past decade [1]. The ability for calcifying organisms to form skeletons will likely be reduced and many species and communities will experience net loss of CaCO_3_ [2], [3], [4] when pH and saturation states (Ω aragonite or calcite) fall to predicted levels over the next 100 years [5], [6]. Coral reefs are among the marine ecosystems most vulnerable to declining pH and Ω as the corals and crustose coralline algae (CCA), which deposit CaCO_3_ and build structurally complex habitats, support extraordinary levels of biodiversity [7], [8]. Thus not only could corals and other calcifying organisms suffer directly from OA but the cascading consequences of reef loss to the flora, fauna and human societies dependent on these systems are likely to be substantial [9].

To date, most studies examining the biological consequences of OA have focused on how reduced pH and/or increased pCO_2_ affect the physiological response of single organisms in controlled mesocosms [but see 10]. In general, calcifying species respond negatively to these controlled, static treatment conditions and experience reduced survival, calcification, and growth [1]; severity of response may be related to the polymorph of CaCO_3_ precipitated [11]. However, forecasting ecosystem consequences to projected future acidification based upon hypothesized tolerance limits for particular species is risky for several reasons. First, the shallow coastal species used in each of these laboratory studies inhabit naturally variable nearshore environs. Without a clear understanding of the range of ambient variability in pH and carbonate chemistry across reefs, it is difficult to anticipate relevant biological tolerance ranges or to look for evidence of populations acclimatized and/or adapted to extreme conditions. Further, few studies examine how ecological processes (e.g. community development, species interactions, or shifts in relative abundances and species assemblages) relate to reduced pH, particularly on coral reefs.

Of the few ecologically relevant OA studies that exist for reefs, all indicate that reef-builders have reduced recruitment rates in acid-addition or CO_2_ enrichment experiments [CCA: 10,12,corals: 13,14]. Further, a fleshy seaweed has been shown to outcompete an adult coral under increased pCO_2_ conditions [15]. Because recruitment and survival of reef-building corals and reef-cementing CCA are critical to the resilience of a reef ecosystem in the face of global change, it is imperative that we gain a better understanding of the responses and interactions among multiple taxa to OA [16]. The data that exist to date suggest that commonly reported phase-shifts from dominance by coral to macroalgae may be exacerbated by OA. But without placing the results of these studies within the context of natural environmental heterogeneity of carbonate chemistry it is difficult to predict the outcomes of these interactions and ultimately, the ability for reefs to be resilient.

More informed ‘ocean warming’ experiments have incorporated natural diurnal or seasonal variability recorded in situ into treatment conditions. There are significant ecophysiological consequences of rising and variable temperatures to corals [17] and bivalves [18] not otherwise observed under constantly elevated temperatures. Unlike temperature, ecologically relevant high frequency temporal variability in carbonate chemistry and pH in situ, especially on coral reefs, has been difficult to document until recently [19], thereby limiting our capacity to design more appropriate experiments.

Most OA monitoring efforts on coral reefs have measured total alkalinity (A_T_) and total dissolved inorganic carbon (C_T_) of discrete water samples to constrain the suite of carbonate chemistry parameters. While discrete samples are accurate and precise, they necessarily have low temporal and spatial resolution; consequently, biogeochemical cycling studies have generally been restricted to shallow reef flats or highly impacted, low diversity reefs and sampled over short time scales [e.g. 24–48 hrs; 20,21]. Moored pCO_2_ systems can reveal seasonal patterns of air-sea gas exchange, but have not historically sampled from the reef floor [22], thus disregarding potential feedbacks from reef metabolism and calcification, so relationships between community structure and dynamics and marine chemistry are difficult to elucidate. Despite these limitations, fluctuations in pH, carbonate ion concentration, and saturation state on a high-latitude reef dominated by non-calcifiers have been described with coarse temporal sampling [23]. Peaks in productivity and calcification potentially underlie variability in reef chemistry, but little is known about cumulative effects of high frequency fluctuations in pH on community development and recruitment of early successional reef-building species.

Lack of sufficient temporal and spatial resolution of ambient fluctuations in pH and carbonate chemistry on the benthos creates a critical gap for our understanding of how present day environmental heterogeneity relates to coral reef ecology. Evidence of diel, seasonal and interannual fluctuations in pH make adopting approaches currently used to define temperature anomalies associated with coral bleaching events - e.g. thermal stress anomalies [24] or weekly sea surface temperature anomalies [25] - appealing for monitoring gradual ‘acidification’ that may reduce reef accretion [26]. But given that short-term variability may be as substantial as decadal shifts on reefs, it is unclear if comparable stress anomalies exist for pH, how they should be defined, or if high frequency variability in pH is even relevant to reef accretion and community development. The potential for diel fluctuations, placed within the context of off-shore climatological chemical oceanography, to relate to community assembly and calcification rates on the benthos is considerable, but unknown.

We used autonomous sensors [SeaFETs; 27] to record temperature and pH with high temporal (hourly observations; 7 months of sampling) resolution on the reef benthos (5–10****m depth) at several islands (Kingman, Palmyra and Jarvis) within the newly designated Pacific Remote Island Areas Marine National Monument (PRIMNM) in the central Pacific (Table S1); these islands are uninhabited and lack potentially confounding local impacts (e.g. pollution and overfishing). Although pH and temperature are insufficient to fully constrain the CO_2_ system, they provide previously unavailable insight to ambient environmental variability on remote reefs. Benthic pH values were compared with those from surrounding open-ocean seawater using the only other data available for the region [CO_2_: 28,A_T_: 29], (temperature, salinity, phosphate, and silicate). These climatological data, based on monthly open ocean sampling, represent seawater chemistry values far removed from potential feedbacks from the reef and allowed us to designate regional means and ranges in key parameters based on seasonal amplitude. Each SeaFET sensor was co-located with replicate Calcification/Accretion Units (CAUs) designed to quantify relative species abundances of early successional benthic organisms and net community CaCO_3_ deposition rates (Fig. S1) so we could determine which, if any, metrics of natural variability in benthic pH and temperature were related to community development and reef accretion rates on these remote reefs.

## Materials and Methods

### Study Area

The study was conducted in three of the northern Line Islands that span 7 degrees of latitude crossing the equator and were designated a Marine National Monument in 2009. Kingman Reef and Jarvis Island are uninhabited and although Palmyra Atoll hosts a small research station operated by The Nature Conservancy (<25 people) for part of the year, the level of present day human disturbance is minimal. During WWII, the US Navy used Palmyra as a naval air station and dredged part of the lagoon to join many of the islets with a causeway; these topographical alterations to the atoll are still apparent today. In stark contrast, Kingman Reef has an open exposed lagoon and Jarvis does not have a lagoon. The reefs at all of these locations are considered healthy and lack any present day direct local impacts that plague many other reefs around the world [30]; see percent cover data in Table S1. Sites were selected on the exposed forereef slope (10****m depth) of all 3 islands and additionally, on the shallow, protected western reef terrace of Palmyra (5****m depth). Tidal exchange is highest at −0.15 to 0.8 m and averages >0.9****m and <0.3****m over spring and neap tides, respectively. Instruments and experiments were deployed and retrieved by SCUBA divers from April−October 2010 (Table S1).

### Autonomous and Discrete Observations of pH and Temperature on a Reef Benthos

**Figure 1:**
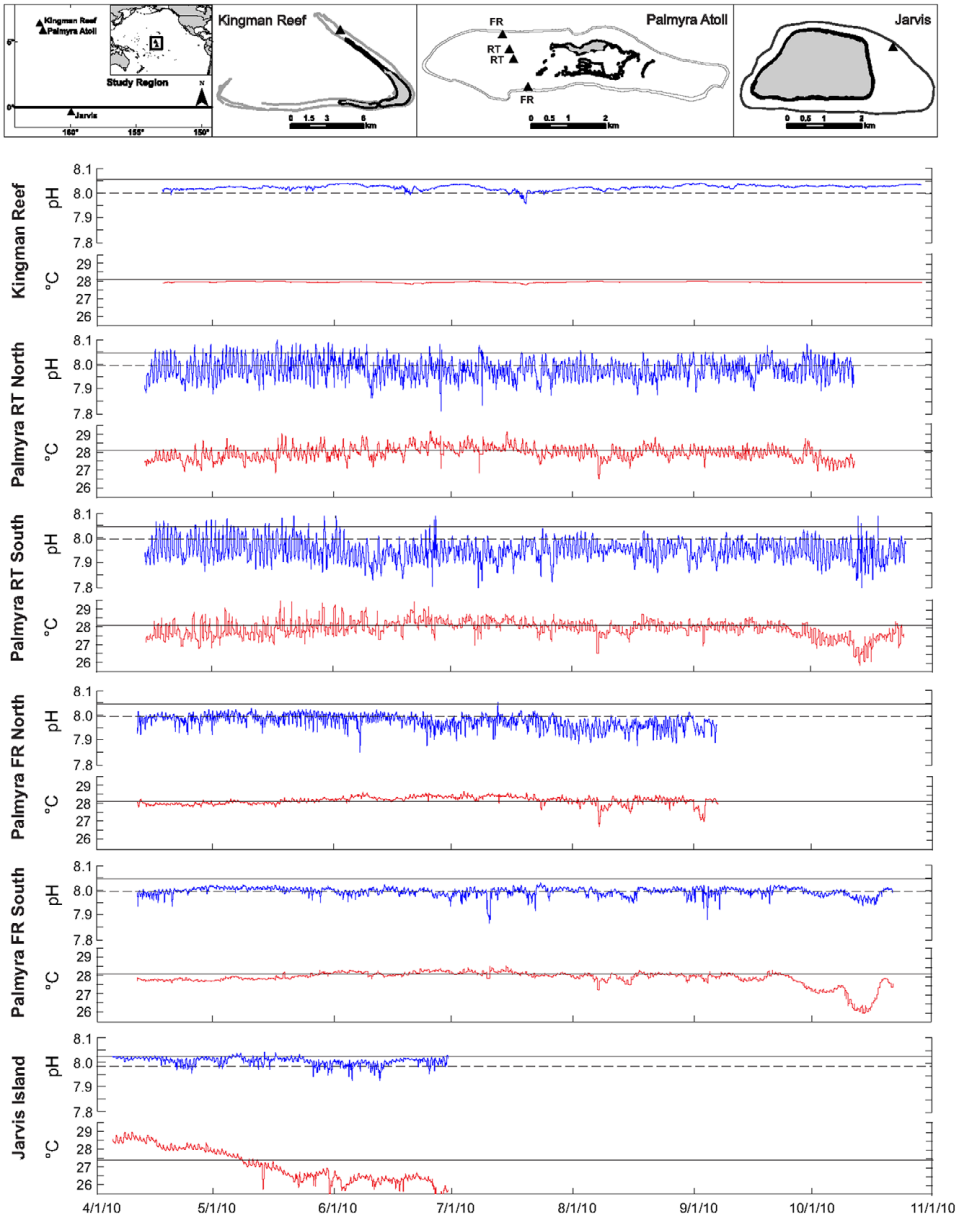
Site locations and continuously logged data (1 hr interval) using the SeaFET sensors. Data are low-pass filtered (period = 2 hrs). The solid black lines in the pH and temperature plots represent a regional climatological mean (e.g. pH_cm_), generated by combining the World Ocean Atlas 2009 (WOA09) data, the Takahashi pCO_2_ climatology [28], and the A_T_ climatology of Lee et al. [29]. In the pH plots, the dashed lines are the pH_csl_ values at the climatological seasonal low. FR = forereef, RT = reef terrace.

SeaFET pH and temperature sensors, autonomous data loggers based on the Honeywell Durafet® pH sensor [27], were affixed to the substrate with cable ties wrapped around the housing and through holes in the reef. In total, 6 SeaFETs were deployed at 3 islands for eight months (Table S1; Fig. 1 see inset maps). Immediately prior to deployment the sensors were calibrated to a discrete sample in a common vessel. The sensors were set to record pH and temperature at 1 hr intervals.

Water samples for total alkalinity (A_T_) and total dissolved inorganic carbon (C_T_) were collected in 500 mL Corning brand pyrex sample bottles and fixed with 200 µL saturated HgCl_2_ solution (1% headspace). To confirm sensor stability and further constrain the carbonate chemistry on the reef, water samples were also taken periodically near the sensors in the field (more frequent sampling was prohibited due to the remoteness of the sites). Water samples were collected adjacent to the pH sensors in Niskin bottles by SCUBA divers and transferred to 500 mL Corning brand pyrex sample bottles, fixed with 200 µL saturated HgCl_2_ solution (1% headspace), and sealed with glass stopper and grease immediately after the divers surfaced. C_T_ and A_T_ were determined in the Dickson lab at Scripps Institution of Oceanography following standard protocols [31] and salinity was estimated from density measured using a Mettler Toledo Model DE45.

Carbon dioxide equilibrium and mineral solubility calculations were performed using CO2SYS (version 14) [32] with constants recommend by Dickson et al. [31]. Analyses were conducted on discrete water samples taken for sensor calibration and on data reported from previous research in the area [33]; pH reported throughout this work is reported on the seawater scale (Tables S2– Table S3, Table S4).

### Net Reef Calcification and Community Structure

To estimate site specific net CaCO_3_ accretion rates and to relate calcification/dissolution to natural variability in pH, the SeaFET sensors were co-located with Calcification/Accretion Units (CAUs) (Fig. S1). A CAU consisted of a pair of roughly sanded PVC plates (10×10 cm) stacked 1 cm apart and plate pairs (*N = *5 per site) were affixed to reef pavement at each site (>0.5 m apart and 10 cm above the substrate) using stainless steel rods and marine epoxy. Immediately after collection, all four surfaces of each CAU were photographed to determine early-successional community structure using the image analysis software PhotoGrid 1.0 (25 stratified random points analyzed per surface); organisms were sorted into ecological functional groups to look for patterns structuring the communities on the benthos and on the CAUs. Plates were then preserved in 8% formalin for subsequent measures of calcification rates.

To quantify the mass of CaCO_3_ accumulated on a CAU, the plates were dried to a constant weight at 60°C and then weighed. Subsequently, CAUs were submerged in 5% HCl for 48 hrs or until all CaCO_3_ had dissolved. The remaining fleshy tissue was scraped onto pre-weighed 11 µm cellulose filter paper, vacuum filtered, dried, and weighed to determine the difference in calcified to fleshy biomass on CAU surfaces. Finally, the acidified, scraped, and dried CAU plates were weighed. Calcimass was determined by subtracting the weight of the fleshy tissue and PVC plates from the total mass of the CAU. For all taxa recruiting to CAUs, the polymorph of CaCO_3_ deposited is known. Thus, the relative net accretion for each polymorph (calcite, aragonite, high Mg calcite) was calculated by multiplying the net calcification rate by the relative abundance of each calcifying taxa of known mineralogy.

### pH Metrics

Temperature and pH dependent calibration coefficients for the SeaFETs were established as described in Martz et al. [27]. Diurnal periodicity was presumed to be the predominant temporal cycling process in the data set, which was too short to resolve annual trends and episodic events. After spectral analysis confirmed this assumption, each time series of pH and temperature was passed through a 2 hr low pass filter to smooth the data, followed by a 32 hr high pass filter to remove low amplitude trends and episodic events before using a 24 hr moving window to calculate daily values for mean and amplitude (as the observed maximum – minimum values for each 24 hr window). The time step in all analyses was 1 hr, corresponding to the sampling interval. To place the nearshore benthic diurnal variability in a regional context, we also found a pelagic climatological mean (pH_cm_) and a climatological seasonal low pH (pH_csl_ = pH_cm_ – seasonal amplitude) that were each estimated for the surrounding offshore seawater of each island (or atoll). The sensor and climatological data were combined into a single pH metric that summed the hourly magnitude of pH above (or below) the seasonal minimum each day (e.g. ∑ pH·hrs above pH_csl_).

The pH climatology used to define a regional offshore pH_cm_ (and seasonal amplitude) was generated by combining the World Ocean Atlas 2009 (WOA09) data for sea surface temperature, salinity, phosphate, and silicate (NODC,http://www.nodc.noaa.gov) with the Takahashi pCO_2_ climatology [28] (CDIAC, http://cdiac.ornl.gov) and the total alkalinity climatology of Lee et al. [29] as reported by CDIAC. The monthly climatology data were re-gridded onto a common 4°×5° (latitude × longitude) map, corresponding to the resolution of the pCO_2_ climatology, and regional pH_csl_ were calculated for each island (or atoll; Table S3). The pCO_2_ climatology for the reference year 2000 was corrected to 2010 using the global average rise of 1.5****µatm yr^−1^ [28].

A Bray-Curtis similarity matrix, with a dummy variable to account for partly denuded assemblages in some replicates [34], was used to look for differences in the spatial distribution of early successional functional groups across sites using CAUs as replicates. Percent cover data were subjected to a dispersion-weighting pre-treatment transformation (functional groups were differentially weighted on the basis of their observed variability in replicate samples). Non-metric Multidimensional Scaling (nMDS) [35] and Canononical Analysis of Principal Coordinates (CAP analyses) [36], [37] showed the same pattern, suggesting the maximum variability calculated by nMDS was due to the influence of our a priori defined factor (site). Thus, CAP analyses and allocation successes are reported; CAP was based on 10,000 permutations [38]. Benthic community patterns were formally tested across sites using PERMANOVA [39], [40] based on unrestricted permutations of the raw data and site as a fixed factor. Linear combinations of the biological (e.g. functional group assemblages on CAUs) and environmental parameters (e.g. temperature and pH metrics) were run to maximize correlations among the canonical coordinate scores.

Community assemblages, percent cover of calcifiers (sub-categorized by polymorph precipitated), and calcification rates of early successional species on CAUs were related to the suite of aforementioned pH metrics. Multivariate regression between pH (or temperature) metrics and calcification rates was not possible because the metrics for pH are not independent (*R* >0.92) and site replication was low. Instead, we used Pearson’s correlations to look for relationships between each pH metric and the mean polymorph-specific calcification data per site (based on *N* = 5 per site, across 6 sites), which were normally distributed (Shapiro-Wilk, P = 0.73).

## Results

The SeaFET data show consistent periodicity in pH associated predominantly with diurnal cycles at each site (Fig. 1), although at least one site also shows evidence of tidally driven semi-diurnal cycle and episodic processes (Fig. S2). The daily amplitude, mean pH, and duration × magnitude above (∑ pH·hrs above pH_csl_) or below the island-specific climatological seasonal low offshore pH was strongly dependent on site (Table S2). Deeper (10****m) fore reef sites experienced significantly less variability in daily pH than the shallow (5****m) reef terrace habitats (t = 6.362, df = 5, P = 0.0055) where pH changed by more than 0.1 units daily (maximum daily range recorded = 0.244). While continuous sampling of total alkalinity (A_T_) and total dissolved inorganic carbon (C_T_) was not possible, periodic discrete sampling confirms that carbonate chemistry on the benthos is highly variable (Table S4).

**Figure 2:**
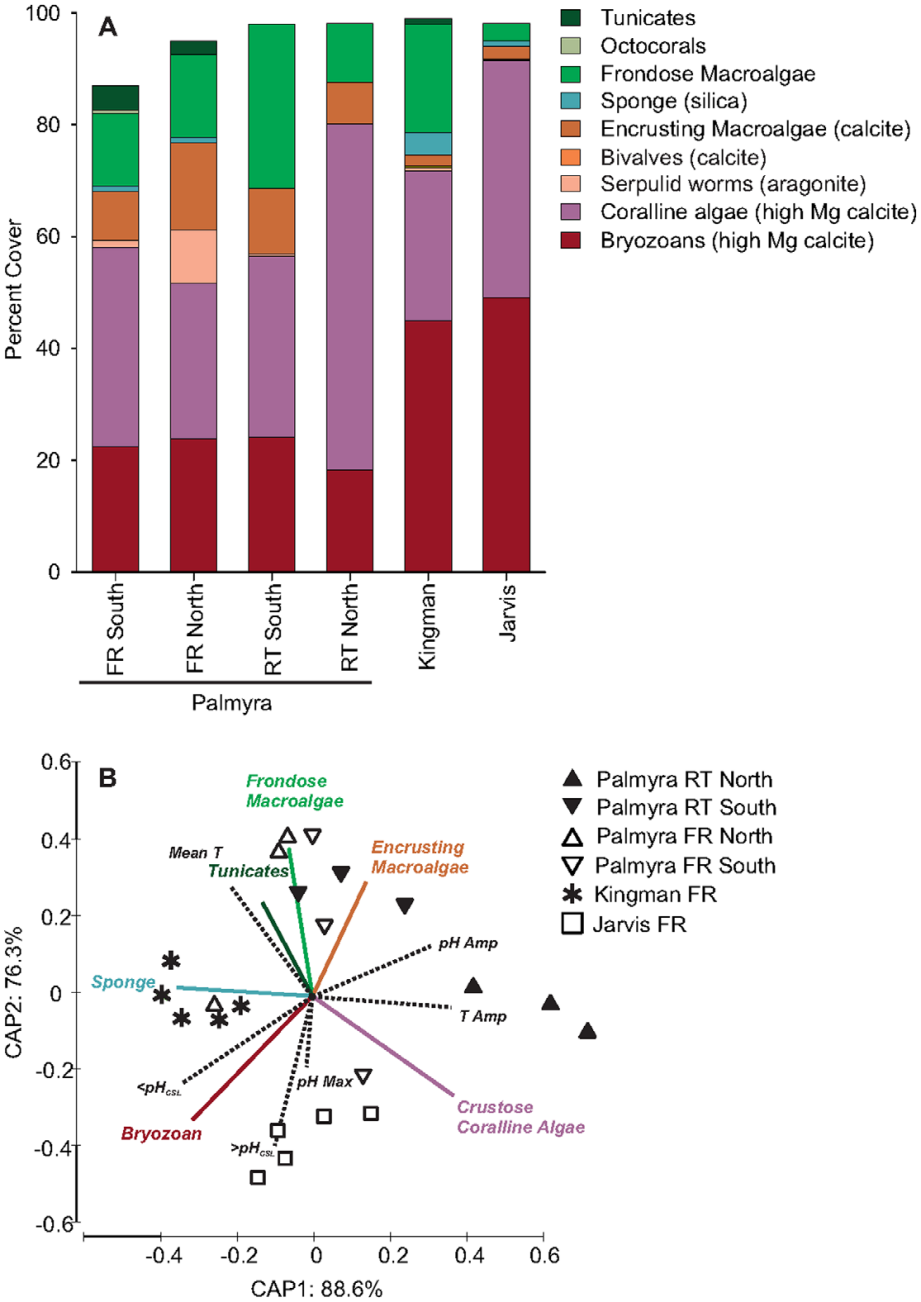
Results of image analyses on Calcification Acidification Units (CAUs). a) Percent cover of the various ecological functional groups. Green bands = ‘fleshy’ invertebrate and algae species. The mineralogy for calcifying species is indicated in the figure legend. b) Original variable vectors of the CAP analysis overlaid as a bi-plot (Spearman Rank correlations >0.5) for both the biological and physio-chemical data from each site. The biological vector colors correspond to functional groups listed in (a).

We recovered a subset of CAUs at the mid-point of deployment (3 months), but found that, on average, more than 20% of the surface remained uncolonized for each CAU. At the final recovery point (7 months), >96% of each CAU hosted biological material. Benthic species assemblages on the CAUs were differentiated by the presence of calcifying and fleshy taxa (CAP analysis, mean allocation success 80%, δ^2^ = 0.886, P = <0.001). Community composition differed significantly among sites (PERMANOVA, Pseudo-F_5,21_ = 9.5113, P<0.0001) and was strongly associated with the pH metrics. Calcifying organisms that dominated CAU surfaces were largely crustose coralline algae (CCA; 36%) and bryozoans (33%), both of which precipitate the more soluble high Mg calcite (MgCO_3_ content 4–40%; Table S6). Percent cover of calcifying organisms increased with the magnitude of ∑ pH·hrs above pH_csl_. Conversely, non-calcified benthic organisms (e.g. fleshy macroalgae, turf algae, sponges, and tunicates; Fig. 2) were more abundant at sites where daily pH was beneath climatological seasonal lows.

Net CaCO_3_ accretion rates of early successional species on CAUs varied within and among islands and were comparable with reef calcification rates measured from the Pacific and Caribbean using chemistry-based approaches (Table S5). Net accretion among sites was positively related to daily ∑ pH·hrs above pH_csl_ (F_5,1_ = 7.778, R = 0.813, P = 0.044).

**Figure 3:**
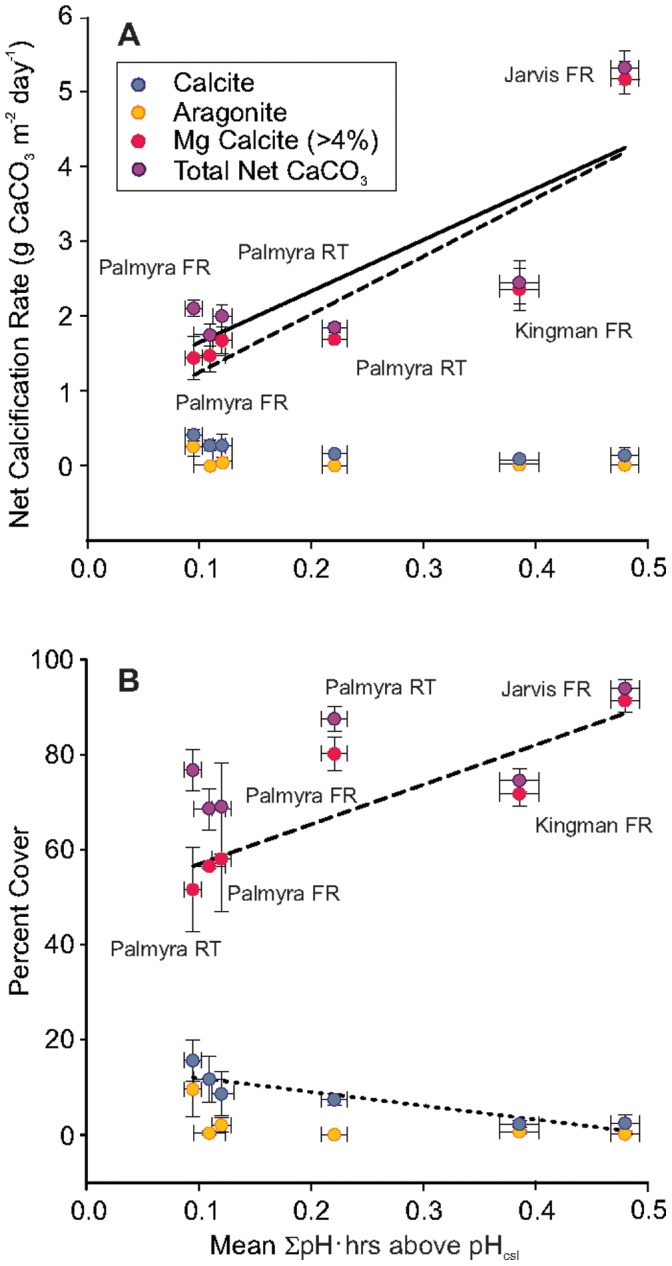
Relationship between net calcification or percent cover analyses on CAUs and relevant pH metrics. a) Means (± SE) of net reef calcification for total CaCO_3_ and each polymorph of CaCO_3_ against mean (± SE) daily ∑ pH·hrs above pH_csl_. Both total net accretion (*Y = *6.86*x* +0.96; *F* = 7.78, *R* = 0.813, P = 0.0444) and high Mg calcite calcification rate (*Y = *7.75*x* +0.47; *F* = 12.26, *R* = 0.868, P = 0.0249) are related to mean daily ∑ pH·hrs above pH_csl_. b) Percent cover of all calcifiers and for each polymorph of CaCO_3_ against mean (± SE) daily ∑ pH·hrs above pH_csl_. Only the percent cover of high Mg calcite is positively related to ∑ pH·hrs above pH_csl_ (*Y = *83.81*x* +48.52; *F* = 12.747, *R* = 0.872, P = 0.0234). Percent cover of calcitic organisms is negatively related to ∑ pH·hrs above pH_csl_ (*Y = *14.85–29.21*x*; *F* = 17.502, *R* = −0.902, P = 0.0139).

When net accretion rates are combined with the percent cover of benthic groups of known mineralogy, we could determine the effect of pH variability on calcification rates for each CaCO_3_ polymorph. We found a strong positive relationship between ∑ pH·hrs above pH_csl_ and the percent cover (F_5,1_ = 12.747, R = 0.872, P = 0.0234) and accretion rate (F_5,1_ = 12.256, R = 0.868, P = 0.0249) of organisms precipitating high Mg calcite (Fig 3, Table S7), but a negative relationship with percent cover of calcitic organisms (F_5,1_ = 8.161, R = -0.820, P = 0.0461). Leverage tests were conducted by calculating Cook’s distances for each point, which were each greater than the fiftieth percentile of the F distribution, indicating that no site had disproportionate influence on the observed relationship.

## Discussion

Ambient variability in both temperature and particularly pH were substantial in the Northern Line islands and oscillated over a diurnal cycle. The observed range in daily pH on reefs encompasses maximums reported from the last century [8.104 in the early evening; 41] to minimums approaching IPCC projected global levels within the next 100 yrs [7.824; 42], values frequently used to represent different treatment levels in experimental manipulations. The daily amplitude in pH measured at Palmyra was similar to that estimated from hourly discrete samples reported there in 1997 [33], but the mean values have dropped by ∼ 0.04 units in the past thirteen years (Table S3). This rate is somewhat higher (∼2×) than predicted by incorporating a global average rise of 1.5****µatm CO_2_ yr^−1^, and may be due to sparse climatological coverage near Palmyra and/or seasonal sampling bias.

Dynamics of pH and seawater chemistry in any coastal ecosystem are driven by co-varying processes, including biological activity, gas exchange, and physical forcing over various time scales (diurnal, seasonal, interannual). Reef metabolism directly affects the CO_2_ system within hydrodynamic boundary layers [43], [44], [45], or just off-shore, as stated in the ‘Coral Reef Ecosystem Feedback’ hypothesis, articulated by Bates *et al.* [23] for seasonal-scale dynamics. Thus species composition and abundance likely also contributed to the spatial variability in the magnitude of benthic diurnal oscillations in pH. However, biogeochemical dissolution and remineralization processes [46], tidal flushing, regional upwelling [47], and oceanographic circulation patterns [48] can dampen, enhance, or swamp biologically driven diurnal fluctuations in pH. Causality cannot be assigned to the spatial patterns in pH variability reported here due to lack of a quantitative hydrodynamic data for these islands. However, it is clear that these remote, uninhabited coral reefs experience highly dynamic fluctuations in nearshore pH that are largely below climatological means estimated from open ocean sampling (Fig. 1, Table S2).

The ecological consequences of OA on coral reefs are as of yet unknown, but natural variation in benthic pH offers a unique opportunity to study scenarios of likely future ocean chemistry. In this study, the relative abundance of competitive fleshy algae and invertebrates increased over that of early successional calcifiers and reef-builders (CCA and bryozoans, respectively) on CAUs across sites that had reduced pH (Fig. 2). This pattern of shifting from dominance by calcifiers to a greater abundance of fleshy species at locations experiencing reduced pH has been observed elsewhere. For instance, benthic communities acclimatized to reduced pH resulting from natural CO_2_ vents host higher bioeroder and fleshy species densities than on adjacent unaffected benthos [26], [49]. Wootton et al. [50] also found calcareous species performed poorly in a temperate tidal pool during ‘low pH years’, and changes in the calcareous nannofossil assemblage during the Paleocene-Eocene thermal maximum [the closest analog in geologic history to current OA; 51] have been attributed to shifts in competitive dominance [52], although the exact mechanisms of how reduced pH altered community structure in either case was unclear. Our data further suggest that community development on these remote uninhabited reefs is strongly related to natural variability in pH, particularly to cumulative and integrative effects of natural diel cycling.

The data presented here from tropical reefs identify daily pH maxima as an important control on calcification. Net accretion among sites was positively related to the magnitude and duration of pH above the climatological seasonal low, despite myriad other ecological (e.g. local supply, species interactions, etc.) and physical oceanographic (e.g. temperature, current magnitude and direction, wave strength, latitudinal gradients, etc.) drivers. In general, accretion rates were higher at sites that experienced a greater number of hours at high pH values each day.

The strength and direction of the relationship between net accretion and naturally varying pH depended upon the polymorph of CaCO_3_ precipitated, despite lack of consistent evidence for this pattern in mesocosm studies [1]. Organisms precipitating more soluble mineral forms of CaCO_3_ are presumed to be less resilient to OA than those precipitating less soluble forms [high Mg calcite vs. calcite and aragonite; 11]. Because the relative content of Mg in shells/skeletons is also positively related to rising temperature [53], these organisms may be particularly susceptible to global change effects including OA [54]. In the Northern Line Islands, where the daily ∑ pH·hrs did not consistently exceed the pH_csl_, the net accretion rate and percent cover of early successional organisms precipitating Mg calcite (i.e. CCA and bryozoans) was lower. A reduction in calcification/growth rates for organisms precipitating Mg calcite may have created space for the calcitic, aragonitic, and non-calcifying species to become competitively dominant. CCA, which precipitate high Mg calcite (>4%), are among the most important reef cementers in the tropics and facilitate coral recruitment. Thus, our results suggest that even small changes in pH could have profound residual and indirect impacts on reef integrity and accretion.

Climatological averaging from discrete offshore sampling may mask potentially important short-term variation in carbonate chemistry and pH in coastal environments, but can provide context for nearshore temporal patterns. Here we show that benthic reef communities are exposed to a wider, and often lower, range of pH values over the diel cycle than predicted by regional seasonal climatology. These novel observations raise the question: what are the relevant metrics of pH to relate to ecological processes expected to be threatened by OA? In the remote central Pacific, the duration and magnitude of benthic pH values above the climatological seasonal low pH correlated strongly with calcification and community structure. Indeed, the more frequently used mean pH, or even the daily maxima or minima, may not be relevant to tolerance limits of organisms or for predicting the ultimate biological impacts of OA. Assigning ecologically relevant tipping points, thresholds, or anomalies for management of OA based solely on open ocean climatological data, as has been done for SST [25], will not be useful without also understanding current relative benthic carbonate chemistry dynamics.

In this study, coral reef communities on remote uninhabited islands in the central Pacific experienced high natural daily variability in pH which corresponded to key differences in net calcification and community development. However, given only six data points and variable physical oceanographic features among islands, this is an initial exploratory, hypothesis generating study, with minimal ability to identify causality. Despite these limitations, these data represent some of the longest and highest resolution field-based observations of natural variability in pH and the associated biological consequences on coral reefs to date. Carbonate chemistry and pH on coral reefs can be highly dynamic and vary significantly within and among sites and islands, which should be considered in future OA mesocosm studies. Finally, our data suggest that as coral reef communities begin experiencing a greater daily duration of low pH values as a result of OA, the abundance of calcified organisms and the structural services they provide will likely be compromised in the foreseeable future.

## Supporting Information

Figure S1 **Design of Calcification/Acidification Unit (CAU) (photo and figure credit: Daniel Merritt, Coral Reef Ecosystem Division, NOAA).**
(TIFF)

Figure S2 **Spectral analyses of time series pH data collected from the SeaFETs. Each plot corresponds to a particular site.**
(TIF)

Table S1 **Deployment metadata for CAUs and SeaFETs in the Northern Line Islands including GPS coordinates, duration of deployment, depths, and mean percent cover (SE) of biological functional groups on the benthos determined from 10 photoquads (analyzed in PhotoGrid.1) taken every 5 m along a randomly placed 50 m transect that follows the pre-designated depth isocline.**
(DOCX)

Table S2 **Mean daily pH metrics (± SE) calculated from the SeaFETs after low pass filtering (period = 2 hrs). Climatological means (pH_cm_) and seasonal lows (pH_csl_) are region specific (see** ***pH metrics*** **[SOM]).** Climatological seasonal lows were calculated as: pH_csl = _pH_cm_ –seasonal amplitude).
(DOCX)

Table S3 **Decadal variation in pH and temperature on the south shore of Palmyra.** Comparison of daily values in 2010 on Palmyra recorded on SeaFETs with discrete water samples taken hourly for 24 hrs in 1997 [33]. Data are means ± SD.
(DOCX)

Table S4 **A summary of seawater (SW) chemistry at 20°C: salinity, total alkalinity (A_T_), pH_SW_, Ω calcite (Ca) and aragonite (Ar), and inorganic carbon content in the discrete bottle samples collected as calibration points for the SeaFETs during deployment.** The values for pH_SW_, pCO_2_, CO_2_, HCO_3_^−^, CO_3_^2−^, Ω Ca, and Ω Ar were calculated from measured values of total carbon (C_T_) and total alkalinity (A_T_) by the computer program CO2SYS (version 14). Data are means (SE) if triplicate bottles were taken.
(DOCX)

Table S5 **Comparison of reef net calcification rates across the Central Pacific from this and other published studies (field estimates using either control volume or total alkalinity anomaly method).**
(DOCX)

Table S6 **Mean (± SE) percent cover of functional groups present on CAUs at each study site. For each functional group, the polymorph of CaCO_3_ precipitated is also reported.**
(DOCX)

Table S7 **Pearson correlation coefficients between CaCO_3_ polymorph calcification rates or percent cover measured on CAUs versus six pH metrics and four temperature metrics measured with SeaFETs at the all reef sites.**
(DOCX)
