# Supplementary figures and images for: Diel Variability in Seawater pH Relates to Calcification and Benthic Community Structure on Coral Reefs
Source: PLoS One. 2012 Aug 28;7(8):e43843. doi: 10.1371/journal.pone.0043843 (PMC3429504; doi:10.1371/journal.pone.0043843)

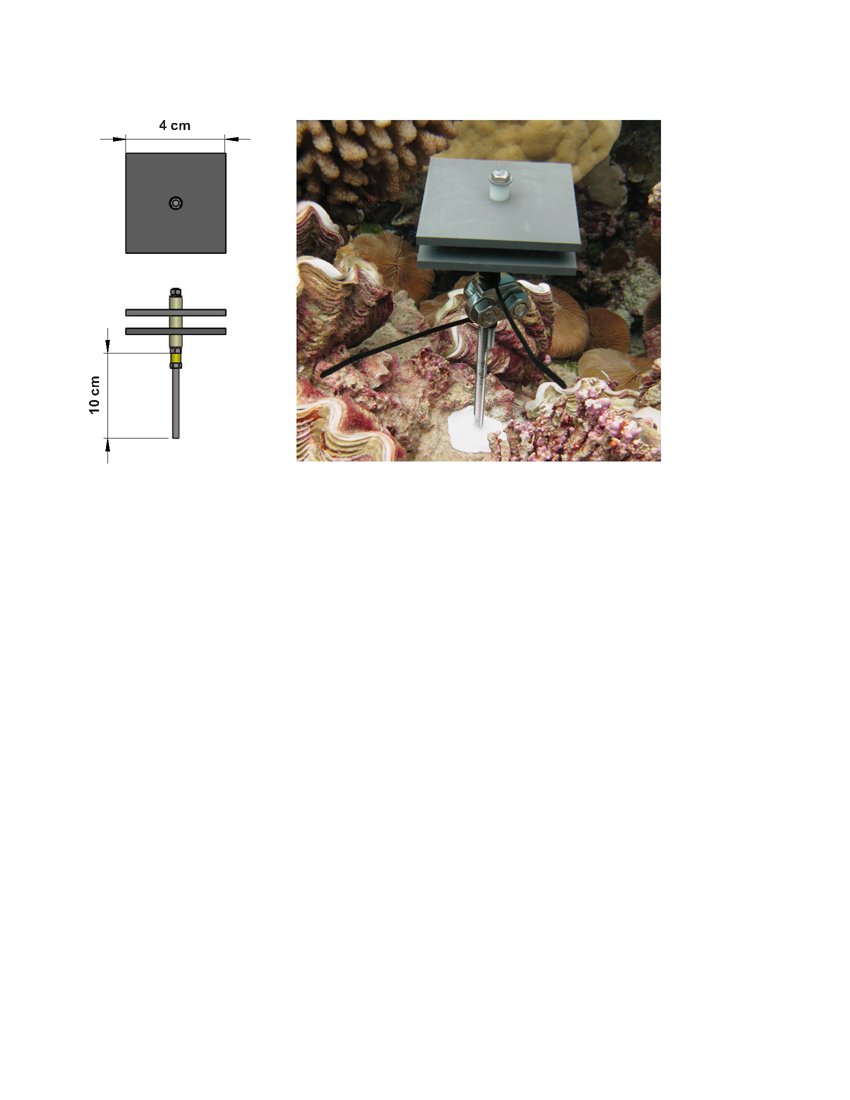

Supplement: Figure S1 — Design of Calcification/Acidification Unit (CAU) (photo and figure credit: Daniel Merritt, Coral Reef Ecosystem Division, NOAA). (TIFF) [file pone.0043843.s001.tiff]

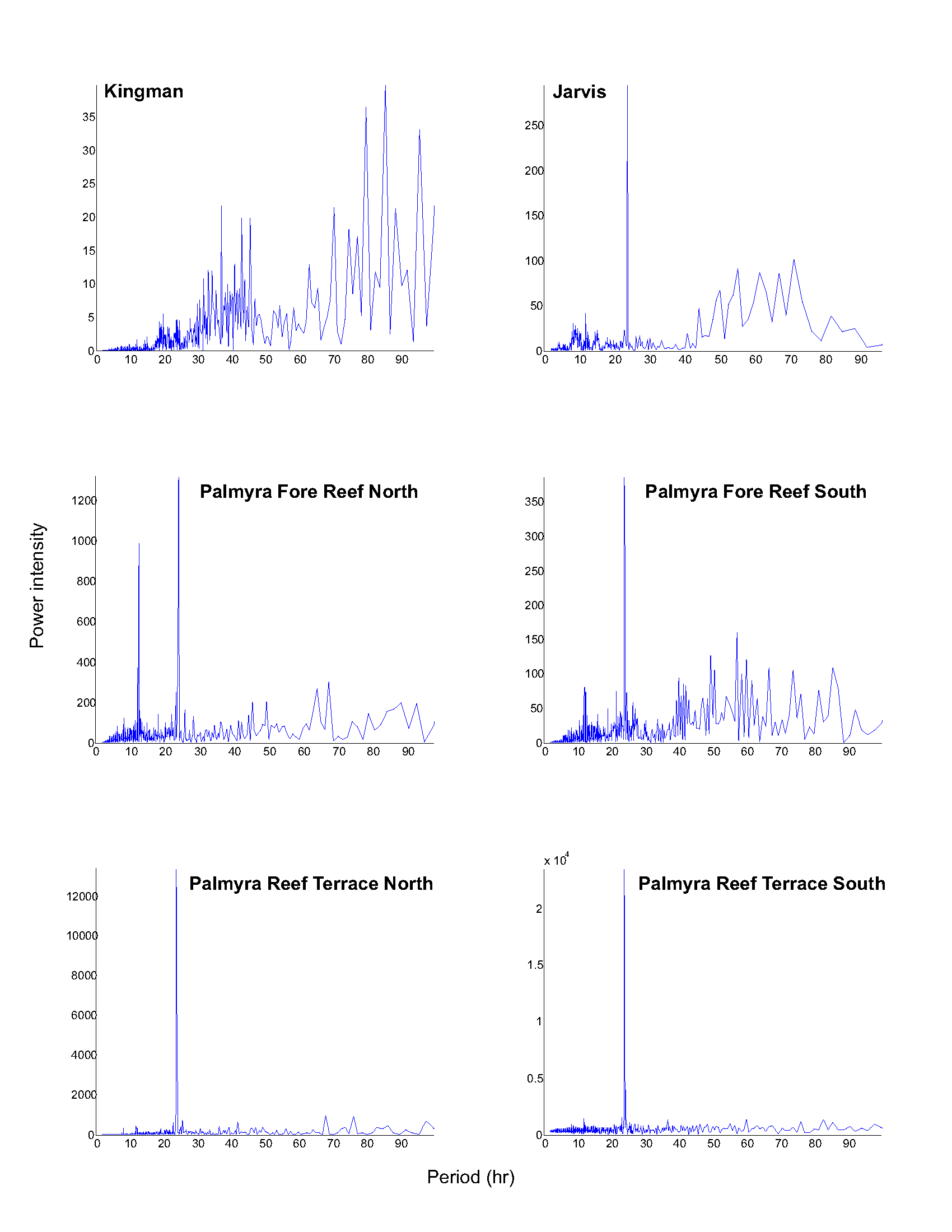

Supplement: Figure S2 — Spectral analyses of time series pH data collected from the SeaFETs. Each plot corresponds to a particular site. (TIF) [file pone.0043843.s002.tif]
