# Supplementary material for: Diel Variability in Seawater pH Relates to Calcification and Benthic Community Structure on Coral Reefs
Source: PLoS One. 2012 Aug 28;7(8):e43843. doi: 10.1371/journal.pone.0043843 (PMC3429504; doi:10.1371/journal.pone.0043843)
Supplement: Table S1 — Deployment metadata for CAUs and SeaFETs in the Northern Line Islands including GPS coordinates, duration of deployment, depths, and mean percent cover (SE) of biological functional groups on the benthos determined from 10 photoquads (analyzed in PhotoGrid.1) taken every 5 m along a randomly placed 50 m transect that follows the pre-designated depth isocline. (DOCX) [file pone.0043843.s003.docx]

**Table S1**. Deployment metadata for CAUs and SeaFETs in the Northern Line Islands including GPS coordinates, duration of deployment, depths, and mean percent cover (SE) of biological functional groups on the benthos determined from 10 photoquads (analyzed in PhotoGrid.1) taken every 5m along a randomly placed 50m transect that follows the pre-designated depth isocline.

| Site | Habitat type | Latitude | Longitude | Depth (m) | Dates Deployed/Recovered | % Hard Coral | No. Coral Species | % CCA | % Turf Algae | % Macro Algae |
| --- | --- | --- | --- | --- | --- | --- | --- | --- | --- | --- |
| Kingman | slope | 6.43887 | -162.38808 | 10 | 4/13/10 – 10/29/10 | 29.4 (8.5) | 198 | 42.2 (6.5) | 2.8 (1.2) | 10.8 (3.4) |
| Palmrya (N) | slope | 5.89711 | -162.12831 | 10 | 4/6/10 – 9/7/10 | 31.3 (4.9) | 185 | 45.6 (4.2) | 14.8 (2.4) | 7.5 (1.7) |
| Palmrya (N) | terrace | 5.88856 | -162.12433 | 5 | 4/8/10 – 10/12/10 | 60.6 (8.4) | 185 | 3.8 (1.6) | 26.3 (6.4) | 9.1 (1.8) |
| Palmrya (S) | terrace | 5.88283 | -162.12197 | 5 | 4/9/10 – 10/25/10 | 56.4 (5.3) | 185 | 7.9 (1.6) | 22.3 (2.3) | 10.5 (1.3) |
| Palmrya (S) | slope | 5.86648 | -162.11361 | 10 | 4/6/10 – 10/22/10 | 50.4 (2.7) | 185 | 17.5 (2.4) | 18.1 (2.9) | 11.8 (2.2) |
| Jarvis | slope | -0.36783 | -159.97953 | 10 | 3/31/10 – 11/4/10* | 13.7 (2.0) | 65 | 34.4 (2.8) | 35.7 (7.8) | 16.1 (2.5) |

*The SeaFET on Jarvis did not record after 7/1/2010
