# Supplementary material for: Diel Variability in Seawater pH Relates to Calcification and Benthic Community Structure on Coral Reefs
Source: PLoS One. 2012 Aug 28;7(8):e43843. doi: 10.1371/journal.pone.0043843 (PMC3429504; doi:10.1371/journal.pone.0043843)
Supplement: Table S2 — Mean daily pH metrics (± SE) calculated from the SeaFETs after low pass filtering (period = 2 hrs). Climatological means (pHcm) and seasonal lows (pHcsl) are region specific (see pH metrics [SOM]). Climatological seasonal lows were calculated as: pHcsl = pHcm –seasonal amplitude). (DOCX) [file pone.0043843.s004.docx]

**Table S2.** Mean daily pH metrics (± SE) calculated from the SeaFETs after low pass filtering (period = 2hrs). Climatological means (pH_cm_) and seasonal lows (pH_csl_) are region specific (see *pH metrics* [SOM]). Climatological seasonal lows were calculated as: pH_csl_ = pH_cm_ –seasonal amplitude).

| Site | SeaFET Daily Means | | | | | | Climatological Annual Means for 2010 | | |
| --- | --- | --- | --- | --- | --- | --- | --- | --- | --- |
|  | pH_benthic_ | pH maximum | pH minimum | pH amplitude | ∑ pH·hrs above pH_csl_ | ∑ pH·hrs below pH_csl_ | pH_cm_ | Seasonal amplitude | pH_csl_ |
| Palmyra Terrace North | 7.981 (0.020) | 8.031 (0.030) | 7.926 (0.024) | 0.106 (0.031) | 0.221 (0.199) | 0.606 (0.343) | 8.049 (0.018) | 0.035 | 8.014 |
| Palmyra Terrace South | 7.958 (0.022) | 8.017 (0.056) | 7.901 (0.041) | 0.115 (0.084) | 0.110 (0.157) | 1.041 (0.438) | 8.049 (0.018) | 0.035 | 8.014 |
| Palmyra Forereef North | 7.982 (0.019) | 8.007 (0.016) | 7.944 (0.028) | 0.063 (0.023) | 0.095 (0.108) | 0.438 (0.368) | 8.049 (0.018) | 0.035 | 8.014 |
| Palmyra Forereef South | 7.995 (0.012) | 8.007 (0.010) | 7.979 (0.021) | 0.028 (0.018) | 0.121 (0.120) | 0.149 (0.186) | 8.049 (0.018) | 0.035 | 8.014 |
| Kingman Reef | 8.025 (0.009) | 8.028 (0.008) | 8.021 (0.012) | 0.007 (0.006) | 0.386 (0.190) | 0.012 (0.065) | 8.054 (0.015) | 0.030 | 8.024 |
| Jarvis Island | 8.005 (0.013) | 8.019 (0.015) | 7.985 (0.030) | 0.034 (0.036) | 0.479 (0.247) | 0.049 (0.097) | 8.023 (0.035) | 0.024 | 7.999 |
