# Supplementary material for: Diel Variability in Seawater pH Relates to Calcification and Benthic Community Structure on Coral Reefs
Source: PLoS One. 2012 Aug 28;7(8):e43843. doi: 10.1371/journal.pone.0043843 (PMC3429504; doi:10.1371/journal.pone.0043843)
Supplement: Table S3 — Decadal variation in pH and temperature on the south shore of Palmyra. Comparison of daily values in 2010 on Palmyra recorded on SeaFETs with discrete water samples taken hourly for 24 hrs in 1997 [33]. Data are means ± SD. (DOCX) [file pone.0043843.s005.docx]

**Table S3.** Decadal variation in pH and temperature on the south shore of Palmyra. Comparison of daily values in 2010 on Palmyra recorded on SeaFETs with discrete water samples taken hourly for 24 hrs in 1997 [33]. Data are means ± SD.

| Time Period | Data Source | pH | | | | Temperature °C | |
| --- | --- | --- | --- | --- | --- | --- | --- |
|  |  | Daily Mean | Daily Maximum | Daily Minimum | Daily Amplitude | Daily Mean | Daily Amplitude |
| 11/21/1997 to 11/22/1997 | Kim Cobb (hourly discrete samples) | 8.038 (0.009) | 8.062 | 8.024 | 0.038 | 27.87 (0.94) | 2.33 |
| 9/18/2010 to 10/01/2010 | SeaFET (hourly continuous sampling) | 7.999 (0.011) | 8.010 (0.010) | 7.985 (0.017) | 0.025 (0.017) | 27.76 (0.36) | 0.19 (0.09) |
