# Supplementary material for: Diel Variability in Seawater pH Relates to Calcification and Benthic Community Structure on Coral Reefs
Source: PLoS One. 2012 Aug 28;7(8):e43843. doi: 10.1371/journal.pone.0043843 (PMC3429504; doi:10.1371/journal.pone.0043843)
Supplement: Table S4 — A summary of seawater (SW) chemistry at 20°C: salinity, total alkalinity (AT), pHSW, Ω calcite (Ca) and aragonite (Ar), and inorganic carbon content in the discrete bottle samples collected as calibration points for the SeaFETs during deployment. The values for pHSW, pCO2, CO2, HCO3 −, CO3 2−, Ω Ca, and Ω Ar were calculated from measured values of total carbon (CT) and total alkalinity (AT) by the computer program CO2SYS (version 14). Data are means (SE) if triplicate bottles were taken. (DOCX) [file pone.0043843.s006.docx]

**Table S4**. A summary of seawater (SW) chemistry at 20 °C: salinity, total alkalinity (A_T_), pH_SW_, Ω calcite (Ca) and aragonite (Ar), and inorganic carbon content in the discrete bottle samples collected as calibration points for the SeaFETs during deployment. The values for pH_SW_, pCO_2_, CO_2_, HCO_3_^-^, CO_3_^2-^, Ω Ca, and Ω Ar were calculated from measured values of total carbon (C_T_) and total alkalinity (A_T_) by the computer program CO2SYS (version 14) . Data are means (SE) if triplicate bottles were taken.

| Site | Date Time | Salinity | A_T_ (µmol kg^-1^) | C_T_ (µmol kg^-1^) | pH_SW_ | HCO_3_^-^ (µmol kg^-1^) | CO_3_^2-^ (µmol kg^-1^) | CO_2_ (µmol kg^-1^) | pCO_2_ (µatm) | Ω Ca | Ω Ar |
| --- | --- | --- | --- | --- | --- | --- | --- | --- | --- | --- | --- |
| Palmyra Terrace North | 4/9/2010 22:00* | 35.00 | 2301.20 | 1991.53 | 8.04 | 1760.50 | 220.11 | 10.92 | 396.31 | 5.30 | 3.51 |
|  | 6/27/2010 13:25 | 34.52 | 2183.90 | 1904.56 | 7.95 | 1694.88 | 197.77 | 11.90 | 469.81 | 4.82 | 3.22 |
|  | 7/7/2010 13:32 | 34.69 | 2228.70 | 1933.73 | 7.99 | 1713.56 | 208.88 | 11.29 | 432.77 | 5.07 | 3.38 |
|  | 10/27/201010:00 | 34.76 | 2258.58 | 1975.72 | 7.98 | 1761.49 | 202.05 | 12.18 | 458.16 | 4.89 | 3.25 |
|  | 10/28/201015:30 | 34.89 | 2234.35 | 1909.72 | 8.05 | 1672.38 | 227.58 | 9.76 | 367.60 | 5.51 | 3.66 |
| Palmyra Terrace South | 4/9/2010 22:00* | 35.00 | 2301.20 | 1991.53 | 8.04 | 1760.50 | 220.11 | 10.92 | 396.31 | 5.30 | 3.51 |
|  | 6/25/2010 13:48 | 34.38 | 2229.30 | 1953.24 | 7.93 | 1742.78 | 197.75 | 12.71 | 509.55 | 4.84 | 3.24 |
|  | 7/6/2010 14:11 | 34.56 | 2215.60 | 1947.65 | 7.94 | 1743.26 | 191.56 | 12.83 | 497.14 | 4.66 | 3.11 |
|  | 10/26/201015:30 | 34.92 | 2239.43 | 1952.42 | 7.99 | 1736.87 | 203.78 | 11.77 | 443.03 | 4.93 | 3.27 |
|  | 10/27/201010:30 | 34.72 | 2265.14 | 1969.98 | 8.00 | 1748.28 | 210.17 | 11.53 | 433.52 | 5.09 | 3.38 |
| Palmyra Forereef North | 4/6/2010 20:00* | 35.27 | 2352.60 | 2041.15 | 8.04 | 1807.43 | 222.52 | 11.20 | 394.89 | 5.34 | 3.52 |
|  | 4/7/2010 14:32 | 34.93 | 2280.68 | 1975.28 | 8.00 | 1746.78 | 217.23 | 11.27 | 430.19 | 5.25 | 3.50 |
|  | 7/3/2010 15:56 | 34.52 | 2242.70 | 1927.70 | 8.01 | 1694.59 | 222.62 | 10.49 | 410.48 | 5.42 | 3.62 |
|  | 7/12/2010 12:31 | 34.39 | 2263.90 | 1958.13 | 8.01 | 1729.63 | 217.50 | 11.00 | 420.87 | 5.29 | 3.52 |
|  | 10/27/201015:30 | 34.75 | 2259.98 | 1958.61 | 8.01 | 1733.57 | 213.90 | 11.14 | 419.00 | 5.18 | 3.44 |
| Palmyra Forereef South | 4/6/2010 20:00* | 35.27 | 2352.60 | 2041.15 | 8.04 | 1807.43 | 222.52 | 11.20 | 394.89 | 5.34 | 3.52 |
|  | 4/8/2010 10:34 | 34.94 | 2284.02 | 1982.94 | 7.99 | 1756.83 | 214.57 | 11.54 | 439.81 | 5.19 | 3.45 |
|  | 6/30/2010 11:41 | 34.64 | 2258.80 | 1966.86 | 7.98 | 1746.79 | 208.25 | 11.82 | 456.11 | 5.06 | 3.37 |
|  | 7/9/2010 15:40 | 34.67 | 2260.60 | 1956.92 | 8.00 | 1730.11 | 215.68 | 11.12 | 425.25 | 5.23 | 3.48 |
|  | 10/26/201012:12 | 34.85 | 2280.46 | 2022.85 | 7.93 | 1821.99 | 186.74 | 14.11 | 531.26 | 4.52 | 3.00 |
|  | 10/28/201010:00 | 34.80 (0.06) | 2282.71 (0.57) | 1975.52 (0.56) | 8.01 (0.0) | 1746.00 (0.94) | 218.45 (0.50) | 11.07 (0.03) | 416.65(0.93) | 5.28 (0.01) | 3.51 (0.01) |
| Kingman Reef | 4/14/2010 20:00* | 35.00 | 2317.90 | 2020.09 | 8.10 | 1798.39 | 210.94 | 10.76 | 333.19 | 5.04 | 3.27 |
|  | 4/15/2010 15:53 | 34.81 | 2281.42 | 1963.31 | 8.03 | 1727.20 | 225.53 | 10.58 | 402.59 | 5.46 | 3.63 |
|  | 10/28/20107:30 | 34.54 | 2235.49 | 1954.87 | 8.01 | 1743.98 | 199.29 | 11.60 | 412.03 | 4.81 | 3.17 |
| Jarvis Island | 3/31/2010 18:30* | 36.15 | 2430.20 | 2151.68 | 8.04 | 1937.09 | 201.29 | 13.31 | 414.64 | 4.76 | 3.10 |
|  | 4/2/2010 15:41 | 35.48 | 2329.84 | 2020.98 | 7.98 | 1788.25 | 220.90 | 11.84 | 459.74 | 5.32 | 3.56 |
|  | 11/12/201012:45 | 35.49 (0.00) | 2325.78 (1.09) | 2074.39 (0.38) | 7.95 (0.0) | 1876.93 (0.07) | 182.80 (0.49) | 14.66 (0.04) | 512.93 (1.40) | 4.37 (0.01) | 2.88 (0.01) |
|  | 11/14/20109:55 | 35.42 (0.02) | 2322.79 (0.32) | 2070.17 (0.84) | 7.95 (0.0) | 1872.12 (1.21) | 183.53 (0.45) | 14.51 (0.06) | 507.45 (2.07) | 4.39 (0.01) | 2.89 (0.01) |

***** Water sample taken from calibration vessel, not from the reef floor.
