# Supplementary material for: Diel Variability in Seawater pH Relates to Calcification and Benthic Community Structure on Coral Reefs
Source: PLoS One. 2012 Aug 28;7(8):e43843. doi: 10.1371/journal.pone.0043843 (PMC3429504; doi:10.1371/journal.pone.0043843)
Supplement: Table S5 — Comparison of reef net calcification rates across the Central Pacific from this and other published studies (field estimates using either control volume or total alkalinity anomaly method). (DOCX) [file pone.0043843.s007.docx]

**Table S5.** Comparison of reef net calcification rates across the Central Pacific from this and other published studies (field estimates using either control volume or total alkalinity anomaly method).

| Method | Location | Net Calcification Rate  (g CaCO_3_ m^-2^ day^-1^) |
| --- | --- | --- |
| *Control Volume* |  |  |
|  | Molokai reef flat [55,56] | -0.10 |
|  | Biscayne Bay [55] | 0.07 |
| *CO_2_ flux* |  |  |
|  | Fanning Island [57] | 2.74 |
|  | One Tree Island [58] | 4.11 |
|  | Lizard Island [59] | 4.93 |
|  | Kaneohe Bay [60] | 7.12 |
| *Total Alkalinity Anomaly* |  |  |
|  | Canton Atoll [61] | 1.37 |
|  | Johnston Atoll [62] | 3.01 |
|  | Enewetak Atoll [63] | 4.11* |
|  | Northern Gulf of Aqaba [64] | 5.00 |
|  | Okinawa [65] | 5.43 |
|  | Lizard Island [62] | 7.40 |
| *Calcimass* |  |  |
|  | Palmyra Atoll (this study) | 1.92 |
|  | Kingman Reef (this study) | 2.45 |
|  | Jarvis (this study) | 5.32 |

* This value is reduced to 2.74 when adjusted for independently measured bioerosion rates.
