# Supplementary material for: Diel Variability in Seawater pH Relates to Calcification and Benthic Community Structure on Coral Reefs
Source: PLoS One. 2012 Aug 28;7(8):e43843. doi: 10.1371/journal.pone.0043843 (PMC3429504; doi:10.1371/journal.pone.0043843)
Supplement: Table S6 — Mean (± SE) percent cover of functional groups present on CAUs at each study site. For each functional group, the polymorph of CaCO3 precipitated is also reported. (DOCX) [file pone.0043843.s008.docx]

**Table S6.** Mean (± SE) percent cover of functional groups present on CAUs at each study site. For each functional group, the polymorph of CaCO_3_ precipitated is also reported.

| Functional Group | Mineralogy | Percent Cover | | | | | | |
| --- | --- | --- | --- | --- | --- | --- | --- | --- |
|  |  | Palmyra | | | | Kingman | Jarvis |  |
|  |  | North FR | North RT | South RT | South FR | North FR | East FR |  |
| Bryozoans | 8-10 % Mg [54,66] | 23.87 (9.31) | 18.28 (2.20) | 24.07 (1.46) | 22.39 (6.94) | 44.96 (0.77) | 49.11 (3.34) |  |
| Coralline Algae | 4-32% Mg [53] | 27.73 (1.44) | 61.90 (5.43) | 32.45 (1.98) | 35.67 (4.12) | 26.78 (2.60) | 42.24 (1.57) |  |
| Serpulid Worms | Aragonite | 9.53 (5.66) | 0 | 0.33 (0.33) | 1.32 (0.88) | 0.61 (0.40) | 0.20 (0.20) |  |
| Peyssonnelia spp. | Aragonite [67] | 15.62 (4.31) | 7.35 (1.19) | 11.68 (4.80) | 8.59 (4.63) | 2.02 (0.85) | 2.21 (1.74) |  |
| Bivalves | Calcite | 0 | 0 | 0 | 0 | 0.20 (0.20) | 0.20 (0.20) |  |
| Sponges | Silicate | 1.01 (0.58) | 0 | 0 | 1.01 (0.58) | 4.04 (1.01) | 1.02 (1.02) |  |
| Fleshy Macroalgae |  | 14.85 (2.29) | 10.58 (2.21) | 29.45 (4.77) | 13.01 (4.11) | 19.38 (2.52) | 3.22 (1.16) |  |
| Octocorals |  | 0 | 0 | 0 | 0.67 (0.67) | 0 | 0 |  |
| Tunicates |  | 2.37 (1.23) | 0 | 0 | 4.37 (3.42) | 1.01 (0.55) | 0 |  |
