# Supplementary material for: Diel Variability in Seawater pH Relates to Calcification and Benthic Community Structure on Coral Reefs
Source: PLoS One. 2012 Aug 28;7(8):e43843. doi: 10.1371/journal.pone.0043843 (PMC3429504; doi:10.1371/journal.pone.0043843)
Supplement: Table S7 — Pearson correlation coefficients between CaCO3 polymorph calcification rates or percent cover measured on CAUs versus six pH metrics and four temperature metrics measured with SeaFETs at the all reef sites. (DOCX) [file pone.0043843.s009.docx]

**Table S7.** Pearson correlation coefficients between CaCO_3_ polymorph calcification rates or percent cover measured on CAUs versus six pH metrics and four temperature metrics measured with SeaFETs at the all reef sites.

| Parameter | Daily Metric | Total Net CaCO_3_ | Calcite | Aragonite | Mg Calcite (> 4%) |
| --- | --- | --- | --- | --- | --- |
| *Calcification Rate* | | | | | |
| pH | mean | 0.455 | -0.620 | -0.122 | 0.490 |
|  | maximum | 0.073 | -0.720 | -0.668 | 0.182 |
|  | minimum | 0.439 | -0.533 | -0.076 | 0.465 |
|  | amplitude | -0.422 | 0.341 | -0.080 | -0.422 |
|  | ∑ pH·hrs above pH_csl_ | **0.813*** | -0.706 | -0.481 | **0.868*** |
|  | ∑ pH·hrs below pH_csl_ | 0.541 | -0.412 | 0.033 | 0.544 |
| Temperature (°C) | mean | 0.366 | 0.556 | 0.699 | -0.161 |
|  | maximum | -0.199 | -0.049 | -0.083 | -0.080 |
|  | minimum | 0.476 | 0.467 | 0.609 | -0.039 |
|  | amplitude | -0.395 | -0.314 | -0.419 | -0.016 |
| *Percent Cover* | | | | | |
| pH | mean | 0.248 | -0.754 | -0.146 | 0.454 |
|  | maximum | 0.457 | -0.630 | -0.647 | 0.671 |
|  | minimum | 0.117 | -0.697 | -0.104 | 0.338 |
|  | amplitude | -0.010 | 0.549 | -0.047 | -0.181 |
|  | ∑ pH·hrs above pH_csl_ | 0.678 | **-0.902*** | -0.489 | **0.872*** |
|  | ∑ pH·hrs below pH_csl_ | 0.573 | -0.632 | 0.001 | 0.408 |
| Temperature (°C) | mean | -0.704 | 0.616 | 0.444 | -0.782 |
|  | maximum | -0.563 | 0.675 | 0.279 | -0.669 |
|  | minimum | -0.735 | 0.457 | 0.503 | -0.764 |
|  | amplitude | 0.298 | 0.219 | -0.335 | 0.204 |

* P < 0.05
